# Epigenetic down‐regulation of microRNA‐126 in scleroderma endothelial cells is associated with impaired responses to VEGF and defective angiogenesis

**DOI:** 10.1111/jcmm.16727

**Published:** 2021-06-17

**Authors:** Yongqing Wang, John Sun, Bashar Kahaleh

**Affiliations:** ^1^ Division of Rheumatology and Immunology University of Toledo Medical Center Toledo OH USA; ^2^ University of Chicago Pritzker School of Medicine Chicago IL USA

**Keywords:** angiogenesis, endothelial cells, epigenetics, microRNA‐126, VEGF

## Abstract

Impaired angiogenesis in scleroderma (SSc) is a critical component of SSc pathology. MicroRNA‐126 (miR‐126) is expressed in endothelial cells (MVECs) where it regulates VEGF responses by repressing the negative regulators of VEGF, including the sprouty‐related protein‐1 (SPRED1), and phosphoinositide‐3 kinase regulatory subunit 2 (PIK3R2). MVECs were isolated from SSc skin and matched subjects (n = 6). MiR‐126 expression was measured by qPCR and in situ hybridization. Matrigel‐based tube assembly was used to test angiogenesis. MiR‐126 expression was inhibited by hsa‐miR‐126 inhibitor and enhanced by hsa‐miR‐126 Mimic. Epigenetic regulation of miR‐126 expression was examined by the addition of epigenetic inhibitors (Aza and TSA) to MVECs and by bisulphite genomic sequencing of DNA methylation of the miR‐126 promoter region. MiR‐126 expression, as well as EGFL7 (miR‐126 host gene), in SSc‐MVECs and skin, was significantly down‐regulated in association with increased expression of SPRED1 and PIK3R2 and diminished response to VEGF. Inhibition of miR‐126 in NL‐MVECs resulted in reduced angiogenic capacity, whereas overexpression of miR‐126 in SSc‐MVECs resulted in enhanced tube assembly. Addition of Aza and TSA normalized miR‐126 and EGFL7 expression levels in SSc‐MVECs. Heavy methylation in miR‐126/EGFL7 gene was noted. In conclusion, these results demonstrate that the down‐regulation of miR‐126 results in impaired VEGF responses.

## 1 INTRODUCTION

Systemic sclerosis (scleroderma, SSc) is a chronic autoimmune connective tissue disease that is characterized by autoimmunity, vascular injury, progressive tissue fibrosis and impaired angiogenesis. The pathogenesis of SSc has not yet been fully elucidated; nonetheless, impaired angiogenesis is believed to be a critical component of SSc pathology that occurs despite chronic tissue ischaemia and progressive loss of microvessels.^1^, ^2^, ^3^, ^4^, ^5^ Vascular endothelial growth factor (VEGF or VEGFA) is one of the most potent mediators of both angiogenesis and vasculogenesis.^6^ Ischaemia and hypoxia are the major regulators of VEGF expression through the induction of the transcription factor, hypoxia‐inducible factor (HIF). VEGF binds the type I transmembrane receptor tyrosine kinases VEGFR1 (also called FLT‐1) and VEGFR2 (FLK1/KDR) on endothelial cells, which result in phosphorylation of ERK1/2 MAPK, Akt and p38 MAPK, leading to endothelial cell proliferation and migration.^6^, ^7^, ^8^, ^9^ Several studies have shown that the expression of VEGFA, VEGFR1 and VEGFR2 is markedly up‐regulated in SSc.^10^, ^11^, ^12^, ^13^, ^14^ However, adaptive angiogenesis is absent despite the progressive loss of capillaries.^4^ MiR‐126, encoded by an intron of the EGF‐like domain (EGFL7) gene, is abundantly expressed in the endothelium. MiR‐126 regulates angiogenic signalling by regulating responses to VEGF in MVECs in part by direct repression of negative regulators of the VEGF signalling pathway, including the sprouty‐related EVH1 domain containing 1 (SPRED1) and phosphoinositide‐3 kinase regulator subunit 2 (PIK3R2), which negatively regulate VEGF signalling via the RAF1‐MAP kinase and PI3 kinase pathways, respectively. SPRED1 and PIK3R2 are validated direct targets of miR‐126, and the defective expression of miR‐126 results in diminished responses to VEGF signalling and impaired angiogenesis.^15^, ^16^, ^17^ Decreased expression levels of EGFL7, miR‐126 host gene, were reported in SSc‐MVECs.^18^

In this study, we examined the expression levels of miR‐126 in normal and SSc skin and MVECs. We also investigated the effects of miR‐126 on the gene expression levels of SPRED1 and PIK3R2 and VEGF‐dependent tube formation and migration in normal and SSc‐MVECs. Moreover, we also inspected the effects of epigenetic regulators on miR‐126 gene expression and the promoter DNA methylation status of the miR‐126 gene in SSc‐MVECs.

## 2 MATERIALS AND METHODS

Cell and Cell culture. This study was approved by the University of Toledo Institutional Review Board. A five‐mm skin biopsy was obtained from the forearm of healthy volunteers and patients with diffuse cutaneous SSc (n = 6) after obtaining a signed written consent form. MVECs were purified by CD31 magnetic beads as previously described.^19^, ^20^ The purity of isolated cells was >98% as determined by flow cytometry analysis using PE anti‐human CD31. Cells were used at the 3‐5th passage in the experiments.

Real‐time quantitative RT‐PCR (qRT‐PCR) for miRNA and gene expression. Total RNA was extracted using miRNeasy Mini Kit (Qiagen, Germantown, MD, USA). For mRNA RT‐qPCR, RNA was reverse transcribed with TaqMan RT reagents (Thermo Fisher Scientific, MA, USA). TaqMan gene expression assays of SPRED1, PIK3R2, EGFL7 and 18S rRNA were used to detect target mRNA expression using Applied Biosystem Real‐time PCR System (Thermo Fisher Scientific, MA, USA). For miR RT‐qPCR, TaqMan miR RT kit and TaqMan mature miR‐assays for miR‐126 were used to quantify miR‐126 expression. RNU44 small nuclear RNA (snRNA) was used as the internal control. Relative expression was calculated using the equation 2‐∆∆Ct. Expression levels of miR‐126 were also calculated as a ratio of molecules of miR‐126/1 million of RNU44 molecules for comparing different expression levels in different cell lines.

Fluorescent in situ hybridization (FISH) and immunofluorescent (IF) labelling. FISH and IF were performed as described previously.^21^, ^22^ Expression levels of hsa‐miR‐126 in MVECs identified by positive CD31 in skin biopsies were detected by miRCURY LNA miRNA ISH Optimization Kit 5 (FFPE) (miR‐126) (Qiagen, Germantown, MD, USA). In situ hybridization reaction was used with the DIG‐labelled LNA miR‐126 probe, a locked nucleic acid oligonucleotide probe labelled at both 5′ and 3′ ends with digoxigenin complementary to human miR‐126. Immunologic detection was done with sheep anti‐DIG‐POD, Fab fragments (sigma) for miR‐126 and primary antibody rabbit Anti‐CD31 (Abcam) for labelling endothelial cells by incubating slides overnight at 4◦C. Then, the slides were incubated at room temperature for 1 hour with Anti‐rabbit‐Alexa Fluor 594 (Invitrogen), and LNA miR‐126 probes were labelled with FITC using a tyramide signal amplification (TSA) system (Perkin Elmer). Finally, the slides were mounted with ProLong Gold Antifade Mountant with DAPI (Life Technologies) and visualized under Olympus Box‐Type Fluorescence Imaging Device Unite Model FSX100 (Olympus America Inc, Center Valley, PA, USA). Images were taken at 20 × magnification by a standard fixed objective: 40x, NA 0.85 (optical zooming from 17× to 80×) with fixed parameters for all samples, using FSX‐BSW software (version 02.01; Olympus America Inc, Center Valley, PA, USA). Filters were selected for each fluorescent probe as follows: ex =345 nm, em = 455 nm for DAPI (blue); ex =590 nm, em =617 nm for Alexa Fluor 594n (Red); and ex =495 nm, em =518 nm for FITC (green). The total fluorescence intensity of miR‐126 and the total area of endothelial cell marker CD31 were quantified using NIH ImageJ software (NIH, USA). The endothelial miR‐126 expression levels were expressed as the mean fluorescence intensity which was calculated by the total fluorescence intensity of miR‐126 divided by the total area of endothelial cell marker CD31. Controls consisted of the scramble‐miR negative control probe and the LNA U6 snRNA positive control probe (Qiagen, Germantown, MD, USA).

Matrigel tube assembly assay. Matrigel tube assembly assays were performed as previously described.^5^, ^23^, ^24^ MVECs were planted on Matrigel at 47.5 × 103 cells with and without the addition of VEGF (50 ng/ml) (R&D Systems, Inc Minneapolis, MN, USA). Cells were incubated at 37℃ for 10 hours and then labelled with 2 µg/ml calcein AM. Tube formation was observed under Cytation 5 Cell Imaging Multi‐Mode Microplate Reader (BioTek Instruments, Inc Vermont, USA). Fluorescence images were taken on a 4× magnification phase compatible objective with the colour GFP (excitation 469 nm/emission 525 nm) using Gene5 software (BioTek Instruments, Inc Vermont, USA). The tube length was calculated using NIH ImageJ and expressed in micrometres.

Scratch‐wound healing assay. Cells were cultured on fibronectin‐coated 6‐well plates, starved overnight, and then scratched with 1‐ml pipet tips, with EBM‐2 medium added with and without VEGF 50ng/ml for 24 hours. The migrated cells were observed under Olympus Box‐Type Fluorescence Imaging Device Unite Model FSX100 and photographed at 4.2× magnification with a standard fixed objective: 10x, NA 0.40 (optical zooming to 4.2 (fixed)), using FSX‐BSW software (version 02.01) (Olympus America Inc, Center Valley, PA, USA). The per cent wound closure was measured by ImageJ. The cell migration was expressed as percentage wound closure (total area‐area not occupied by the cells/total area ×100).

Endothelial Cell migration assay. The migration assay was performed using the Corning BioCoat Angiogenesis System‐Endothelial Cell Migration (Corning, NY, USA). MVECs (1 × 105 cells/well) were added to the upper chambers. The lower chamber was loaded with EBM‐2 alone or EBM‐2 with VEGFA 50 ng/ml. After 24 hours at 37℃, cells were labelled with calcein AM. Fluorescence signals were measured by Cytation 5 microplate reader (BioTek, Winooski, USA). Data are shown as fold migration= (mean RFU of cells migrating through membrane towards VEGF/ (mean RFU of cells migrating through the membrane without chemoattractant). RFU: relative fluorescence units.

Transfection/electroporation of microRNA inhibitor and microRNA mimics. For knockdown of miR‐126, control MVECs (0.5 × 106) were electroporated with 1 µg (100 pmol) of miRCURY LNA miR power inhibitor (Qiagen, Germantown, MD, USA) using the Basic Nucleofector Kit for primary mammalian endothelial cells (Lonza Biologics, Portsmouth, NH) by the Amaxa Nucleofector. A miRCURY LNA miR inhibitor control (Qiagen, Germantown, MD, USA) that is similar in sequence length and LNA design with no homology to any known miR or mRNA sequence in the mouse, rat or human genome was used as a negative control. For overexpression of miR‐126, SSc‐MVECs were transfected with 130 pmol of a miRCURY LNA miR‐126 mimic (Qiagen, Germantown, MD, USA). A negative control miR mimic (miRCURY LNA miR mimic negative control, Qiagen, Germantown, MD, USA) with the same design features as the miRCURY LNA miR mimics was used as a negative control.

Western blot analysis. The concentration of protein in cell lysates was determined by the Bradford reagent. 20‐40 µg of protein per sample was separated on 10% SDS‐PAGE gels and transferred to polyvinylidene fluoride membranes. Relative quantification was performed using ImageJ. The antibodies used in Western blots were as follows: AKT, Phospho‐AKT (Ser473), ERK1/2, Phospho‐ERK1/2 (Cell Signaling, Danvers, MA), EGFL7 (Abcam, Cambridge, MA, USA), SPRED1 (Abcam), PIK3R2 (Thermo Fisher, Rockford, IL, USA) and GAPDH (Santa Cruz Biotechnology, Inc Mississauga, ON, Canada).

Bisulphite sequencing. The methylation status of the CpG dinucleotides in the EGFL7 promoter region (45‐523 bps upstream of ATG in EGFL7‐202 gene) was analysed. Genomic DNA was isolated from MVECs using the QIAamp DNA Blood Mini Kit and bisulphite conversion was performed using the EpiTect Bisulfite Kit (Qiagen, Germantown, MD, USA). The fragments of EGFL7 promoter were amplified using the following specific primer pairs designed with the MethPrimer software (https://www.urogene.org/methprimer/): forward, 5′‐ TGAGAAATTAAATTTTAGAAG G GTTGA −3′; reverse, 5′‐ AACACAAAACATAACCCCTAAATCTC −3′. PCR products were gel purified and cloned into the pGEM‐T vector (Promega). Individual bacterial colonies were selected and sequenced using the Sp6 reverse primer (Eurofins Genomics, Louisville, KY, USA) to analyse DNA methylation.

Statistical analysis. For statistical analyses, mean values with standard deviation (s.d.) are shown in most graphs that were generated from several repeats of biological experiments. *P* values were obtained from *t* tests with paired or unpaired samples, with a significance set at *P* < 0.05.

## 3 RESULTS

### 3.1 Abundant miR‐126 expression in control MVECs and reduced expression levels in SSc‐MVECs and skin

**FIGURE 1:**
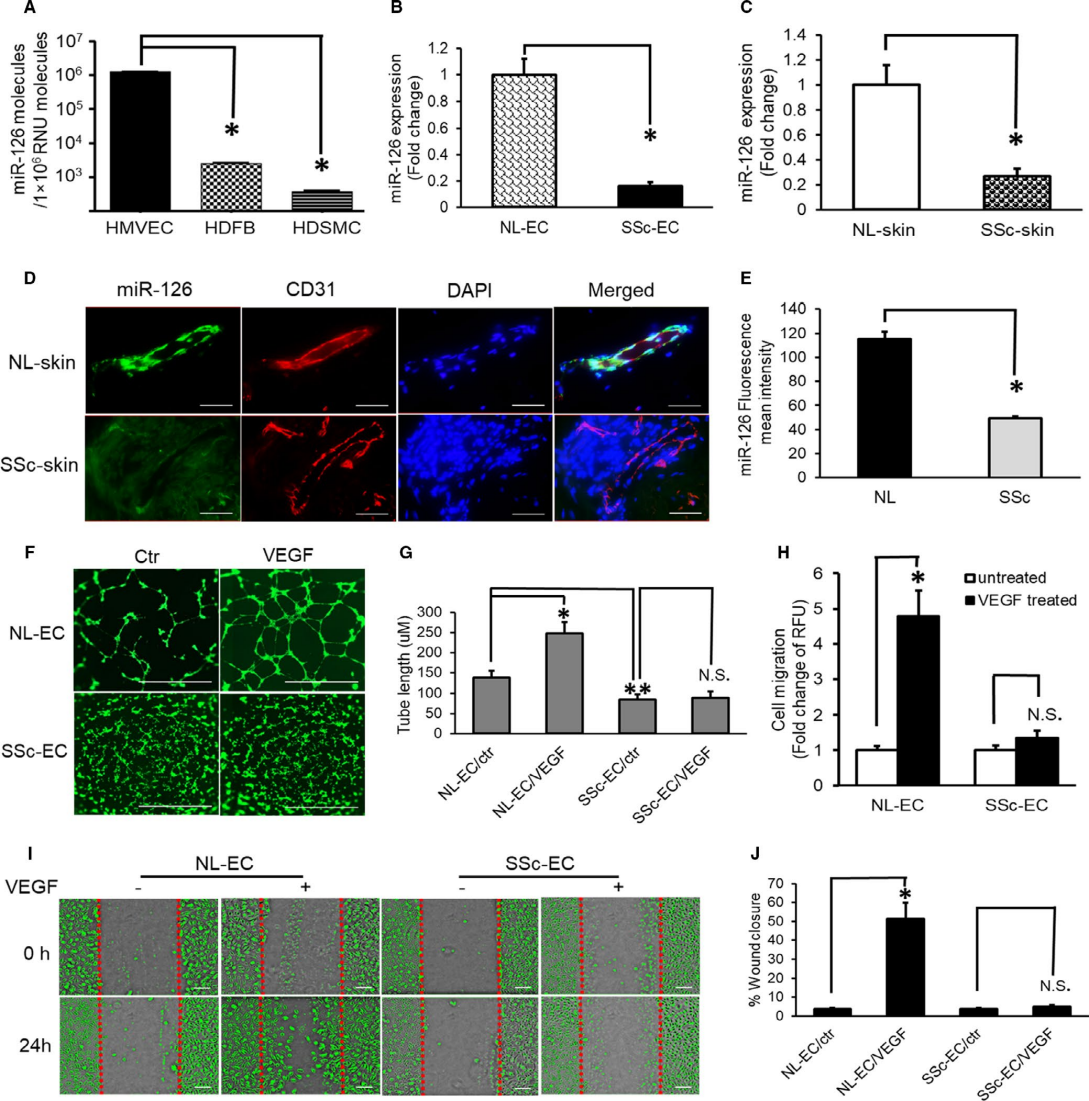
Reduced miR‐126 expression and VEGF‐dependent angiogenesis responses in scleroderma endothelial cells. A, MiR‐126 expression levels in MVECs, fibroblasts (HDFB) and smooth muscle cells (HDSMC) (n = 3 each). B, Decreased miR‐126 expression in SSc‐MVECs (n = 6). C, Reduced expression of miR‐126 in SSc skin (n = 3). D, MiR‐126 staining in normal and SSc skin. Co‐localization of miR‐126 (green) and CD31 (red, MVECs marker). Bar = 100 μm. E, MiR‐126 expression in SSc skin significantly decreased compared to control (n = 3). F and G, VEGF induced tube formation (F). Tube length expressed in micrometres (G). VEGF induced significant increase in tube length in normal MVECs, but failed to do so in SSc‐MVECs (n = 6). Bar = 1000 μm. I‐J, Scratch‐wound assay analysis of NL and SSc‐MVECs response to VEGF expressed as %wound closure. VEGF induced significant wound closure in normal MVECs compared to its untreated control (n = 6), but was unable to induce in SSc‐MVECs. Bar = 200 μm. H, Cell migrations were quantitated using calcein AM labelling. SSc‐MVECs did not exhibit cell migration under VEGF stimulation (n = 6). All values are expressed as mean ±SD. *P < 0.01, labelled group versus the control group. **P < 0.05, labelled group versus NL‐MVECs/ctr. *P* < 0.05 was considered significant

The expression levels of miR‐126 were assessed in control MVECs, control HDFBs (human dermal fibroblasts), control HDSMCs (human dermal smooth muscle cells) and SSc‐MVECs by qPCR. The expression levels of miR‐126 in NL‐MVECs were approximately 500 times higher than in HDFBs and 5000 times higher than in HDSMCs (Figure 1A). The data confirmed that miR‐126 is expressed mainly in MVECs. MiR‐126 expression was significantly down‐regulated in SSc‐MVECs by 0.16 ± 0.03 folds, compared to control (Figure 1B; *P* < 0.01). MiR‐126 expression levels were also examined in freshly isolated RNA obtained from skin biopsies of SSc and control subjects by qPCR (n = 3). Significant reduction in miR‐126 expression was noted in SSc skin by 0.27 ± 0.06 folds compared to control samples (Figure 1C; *P* < 0.01). Moreover, the expression of miR‐126 was examined in paraffin sections of skin biopsies by in situ hybridization followed by quantitative densitometry analysis using ImageJ (Figure 1D‐1E). Co‐localization of miR‐126 and endothelial‐specific marker CD31 was observed in skin biopsies (Figure 1D). Similarly, the miR‐126 expression levels in SSc‐MVECs were also significantly down‐regulated by 2.32 folds in SSc skin paraffin sections when compared to control (Figure 1E; *P* < 0.01).

### 3.2 Diminished SSc‐MVECs VEGF‐dependent angiogenesis responses

To examine the responses of MVECs to VEGF‐induced angiogenesis, NL‐MVECs and SSc‐MVECs were plated onto Matrigel to investigate capillary tube assembly and plated into fibronectin‐coated 6‐well plates and Corning FluoroBlok™ 96‐well insert to test MVEC migration.

For Matrigel assay, MVECs were labelled with calcein AM and observed under a microscope. The capillary morphogenesis was quantified by measuring the total tube segment length. Figure 1F shows tube formation by control and SSc‐MVECs before and after the addition of VEGF. The addition of VEGF to control MVECs resulted in robust tube formation with an average tube length of 246.83 ± 28.69 µM versus 137.49 ± 16.48 µM in unstimulated cells (Figure 1G; *P* < 0.01), whereas almost no responses to VEGF were seen in SSc‐MVECs with the average tube length of 88.58 ± 15.46 µM in VEGF‐treated SSc‐MVECs versus 84.35 ± 12.68 µM in SSc untreated cells (Figure 1G, P >.05). Moreover, the tube length in SSc‐MVECs was also significantly lower than in control MVECs at baseline levels (Figure 1G; *P* < 0.05), which suggests that SSc‐MVECs also have an impaired angiogenesis response to the low amount of growth factors in the Matrigel or to Matrigel itself.

In the scratch test, the addition of VEGF enhanced control MVECs migration (Figure 1I) and resulted in 51.34 ± 8.64% wound closure in 24 hours versus 3.86 ± 0.62% in unstimulated control (Figure 1J; *P* < 0.01), while no significant response was seen in VEGF‐treated SSc‐MVECs with 5.02 ± 0.98% wound closure versus 3.62 ± 0.65% in untreated SSc‐MVECs.

(Figure 1I‐J, P >.05). Similarly, for Corning FluoroBlok endothelial migration assays, VEGF significantly increased cell migration in control MVECs by 4.8 ± 0.72 folds versus baseline values (Figure 1H; *P* < 0.01), whereas SSc‐MVECs with VEGF stimulation exhibited similar migration value to baseline values 1.35 ± 0.2 folds versus 1 ± 0.14 folds in control‐SSc‐MVECs (Figure 1H; *P* >.05).

### 3.3 Reduced miR‐126 expression in SSc‐MVECs is associated with the up‐regulation of SPRED1 and PIK3R2

**FIGURE 2:**
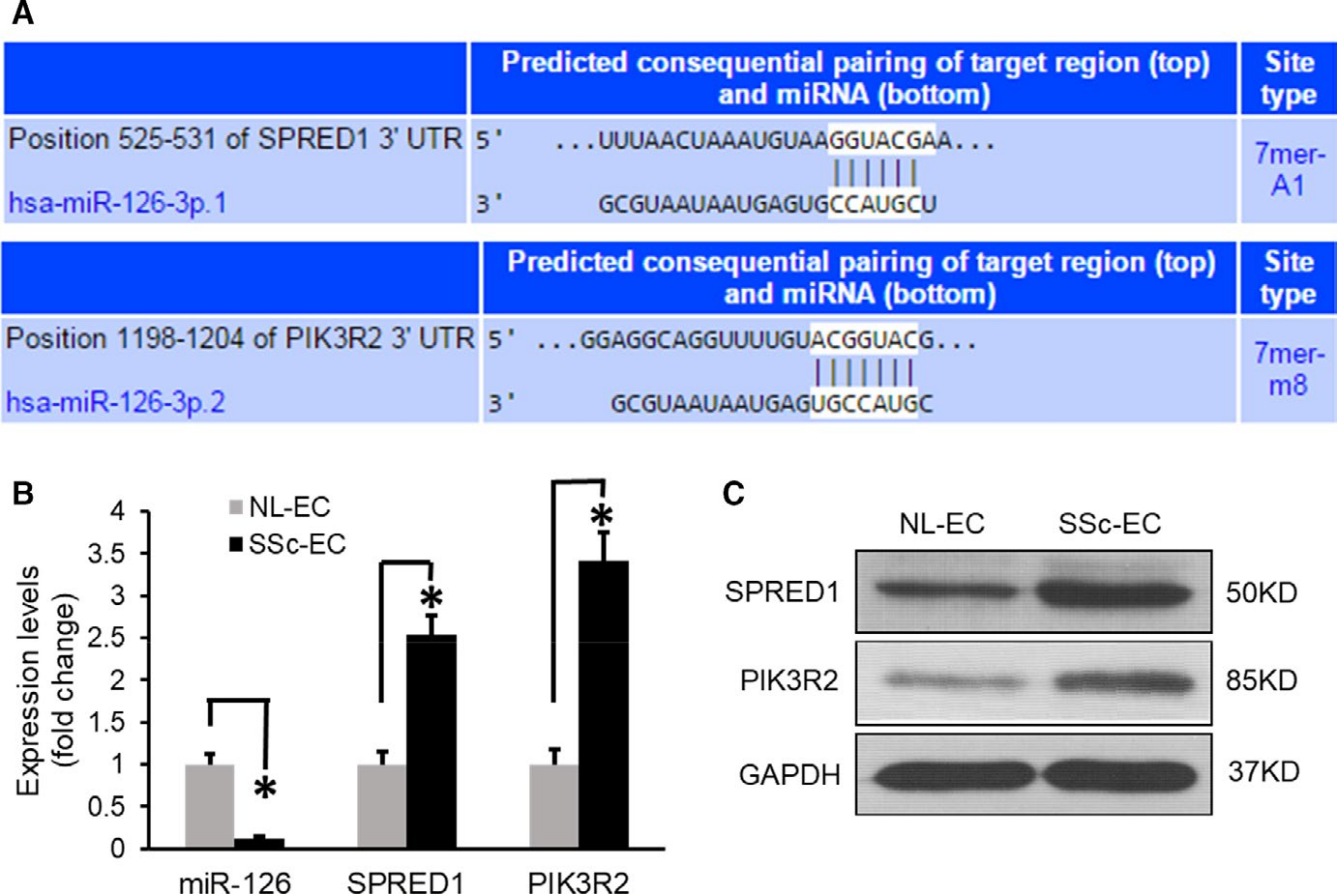
Reduced miR‐126 expression in SSc‐MVECs is associated with up‐regulation of SPRED1 and PIK3R2 expression. A, SPRED1 and PIK3R2 are potential targets of miR‐126 as was predicted by TargetScan. The sequences of the miR‐126 binding site in the 3’‐untranslated region (3’‐UTR) of SPRED1 and PIK3R2 are shown. B, qPCR demonstrates enhanced expression of SPRED1 and PIK3R2 in association with significant underexpression of miR‐126 in SSc‐MVECs. *P < 0.01, labelled group versus to the NL‐MVEC control. n = 6 different cell lines. C, Western blot confirmed that SPRED1 and PIK3R2 are overexpressed in SSc‐MVECs

To explore the role of miR‐126 in the defective VEGF‐dependent angiogenesis in SSc‐MVECs, we searched for potential direct mRNA targets of miR‐126. Using TargetScan, we confirmed that there are sequences of the miR‐126 binding site in the 3’‐untranslated regions (3’‐UTR) of SPRED1 and PIK3R2 (Figure 2A), which are the key angiogenesis regulatory genes for VEGF signalling, as reported by previous studies in mice and zebrafish.^15^, ^16^ Next, we measured mRNA levels of SPRED1 and PIK3R2 by qPCR assay. Increased mRNA expression levels of SRED1 by 2.54 ± 0.22 folds and PIK3R2 by 3.42 ± 0.34 folds in SSc‐MVECs were noted, compared with the normal control (Figure 2B; *P* < 0.01) in association with reduced expression of miR‐126 in SSc‐MVECs (Figure 2B). These data suggested that the reduced miR‐126 expression in SSc‐MVECs is associated with up‐regulation of SPRED1 and PIK3R2 expression (Figure 2B). Western blot analysis also confirmed that conclusion on the protein levels (Figure 2C).

### 3.4 Down‐regulation of miR‐126 expression enhances SPRED1 and PIK3R2 and impairs angiogenesis response to VEGF in normal endothelial cells

**FIGURE 3:**
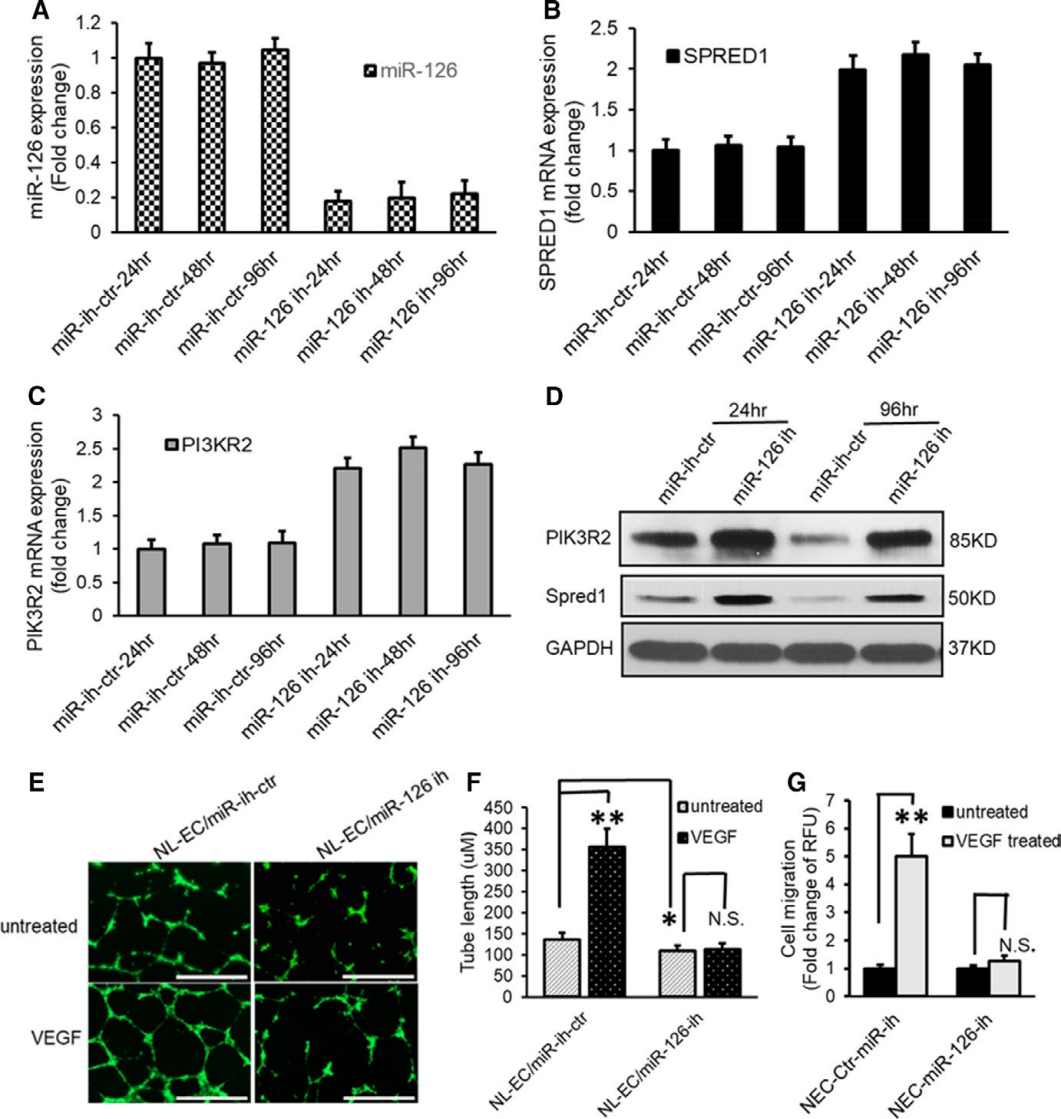
Down‐regulation of miR‐126 enhances SPRED1 and PIK3R2 and impairs angiogenesis response to VEGF in normal endothelial cells. A‐D, MiR‐126, SPRED1 and PIK3R2 expression levels were analysed by qPCR and Western blot after NL‐MVECs were transfected with miR‐126 inhibitor. A, Reduced levels of miR‐126 in NL‐MVECs for up to 96 hours following transfection with miR‐126 inhibitor. B, Enhanced SPRED1 mRNA expression. C, Increased PIK3R2 mRNA expression. D, Increased protein expression levels of SPRED1 and PIK3R2 in NL‐MVECs for up to 96 hours following transfection with miR‐126 inhibitor. E and F, Down‐regulation of miR‐126 associated with decreased tube formation in NL‐MVECs. NL‐MVECs were transfected with miR‐126 inhibitor (miR‐126‐ih) or miR inhibitor negative control (miR‐ih‐ctr). E, Cells were labelled with calcein AM and photographed at 4× magnification. Bar =1000 μm. F, VEGF failed to increase the tube length in miR‐126 knockdown NL‐MVECs. G, Knockdown of miR‐126 expression in NL‐MVECs impaired EC migration response to VEGF. Results are expressed as mean ±SD. n = 6 different cell lines. *P < 0.05, labelled group versus normal MVEC untreated control. **P < 0.01, labelled group versus the other groups

To further validate that the up‐regulation of SPRED1 and PIK3R2 was direct consequence of miR‐126 down‐regulation, we transfected NL‐MVECs with miR‐126 inhibitor or microRNA inhibitor control. MiR‐126, SPRED1 and PIK3R2 expression levels were analysed by qPCR and Western blot, the expression of miR‐126 decreased by 4‐5 folds (Figure 3A), and the mRNA levels of SPRED1 and PIK3R2 were significantly increased by 2.0‐2.2 folds for SPRED1 (Figure 3B) and 2.2‐2.5 folds for PIK3R2 (Figure 3C) relative to negative controls. The protein levels of both SPRED1 and PIK3R2 were also increased by 2 folds (Figure 3D).

To examine the functional consequences of underexpressed miR‐126 on angiogenesis, we examined responses to VEGF in Matrigel tube assembly assay. NL‐MVECs with microRNA control inhibitor (NL‐MVEC/miR‐ih‐ctr) formed complete tubes in response to VEGF stimulation, while NL‐MVECs with down‐regulated miR‐126 failed to form tubes after the addition of VEGF (Figure 3E). The analysis of the tube length showed that the addition of VEGF to NL‐MVEC/miR‐ih‐ctr significantly increased tube assembly with an average tube length of 356.49 ± 42.78 µM versus 136.73 ± 15.67 µM in the untreated control (Figure 3F; *P* < 0.01), while there were no VEGF responses in miR‐126 down‐regulated NL‐MVECs with the average tube length of 112.42 ± 14.61 µM versus 109.35 ± 13.12 µM in NL‐MVEC/miR‐126‐ih untreated (Figure 3F; *P* >.05). Moreover, the length of tube in NL‐MVEC/miR‐126‐ih at baseline was also significantly lower than that in NL‐MVEC/miR‐ih‐ctr (Figure 3F; *P* < 0.05), which suggested that the down‐regulation of miR‐126 in NL‐MVECs also inhibited the tube assembly induced by the low amount of other growth factors or Matrigel itself (Figure 3F). Similarly, NL‐MVECs with knockdown miR‐126 showed impaired migration potential after addition of VEGF with the migration rate at 1.26 ± 0.19 folds versus 1 ± 0.11 folds in control (*P* >.05; Figure 3G).

### 3.5 Overexpression of miR‐126 in SSc‐MVECs repressed the expression levels of SRED1 and PIK3R2 and increased VEGF angiogenesis response

**FIGURE 4:**
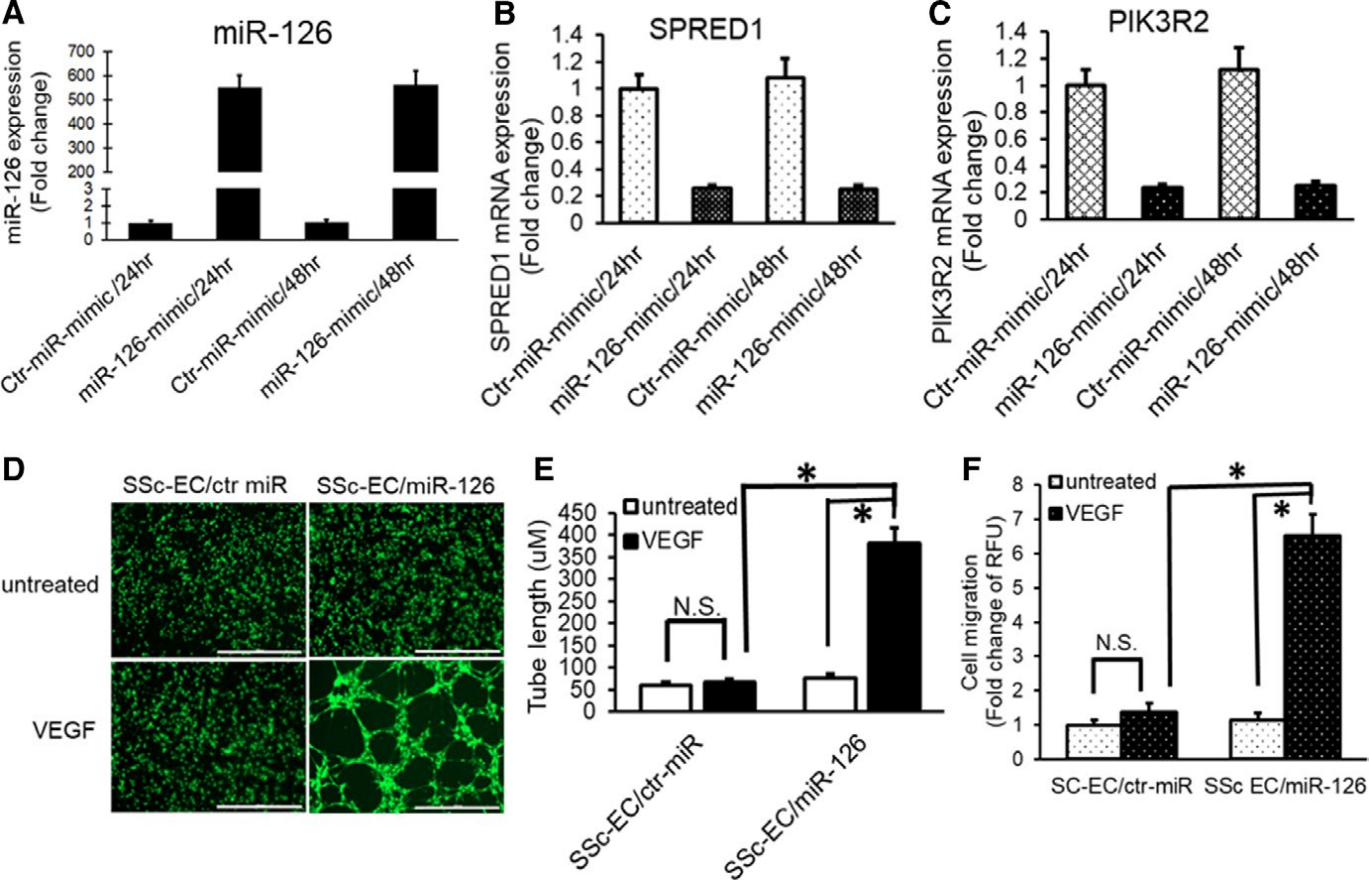
Enhanced expression of miR‐126 in SSc‐MVECs repressed the expression levels of SPRED1 and PIK3R2 and increased VEGF response. A‐C, MiR‐126, SPRED1 and PIK3R2 expression levels were analysed by qPCR after SSc‐MVECs were transfected with miR‐126 mimics. A, Increased levels of miR‐126 in SSc‐MVECs for up to 48 hours following transfection with miR‐126 mimics. B, Decreased SPRED1 mRNA expression. C, Decreased PIK3R2 mRNA expression. D and E, Effects of miR‐126 mimics on tube formation in SSc‐MVECs were examined by Matrigel assay. SSc‐MVECs were transfected with 150 pmol of miR‐126 mimics, and then, the cells were cultured on Matrigel with and without VEGF treatment (50 ng/ml) for 10 hours, labelled with calcein AM and photographed at 4× magnification (D). The capillary tube length was calculated using NIH ImageJ and expressed in micrometres (E). Increase of miR‐126 expression in SSc‐MVECs resulted in up‐regulated VEGF angiogenesis response. There was significant increase in tube length in SSc‐MVECs with overexpression of miR‐126 in response to VEGF. Bar = 1000 μm. F, miR‐126 mimic restored VEGF’s migration response in SSc‐MVECs. Migrated SSc‐MVECs were detected by Corning BioCoat Angiogenesis System: Endothelial Cell Migration. *P < 0.01, labelled group versus the other groups. n = 6 different cell lines

To investigate the effects of overexpression of miR‐126 in SSc‐MVECs on the expression levels of SRED1 and PIK3R2 and angiogenesis responses to VEGF, SSc‐MVECs were transfected with miR‐126 mimics or negative control miR mimic. The expression levels of miR‐126, SPRED1 and PIK3R2 were measured by TaqMan qPCR. Consistent with the data obtained from miR‐126 inhibitor, transfecting a miR‐126 mimic into SSc‐MVECs induced a 550‐560‐fold increase in miR‐126 after 24 to 48 hours transfection (Figure 4A) and significantly decreased the mRNA expression levels of SPRED1 by 0.26‐0.25 folds and PIK3R2 by 0.24‐0.25 folds, compared to control miR mimic (Figure 4B‐C). Moreover, tube assembly formation assay analysis showed that overexpression of miR‐126 in SSc‐MVECs increased VEGF angiogenic response (Figure 4D), with significantly increased tube length to 380.71 ± 36.24 µM from 66.73 ± 8.16 µM in SSc‐MVECs/ctr‐miR/VEGF (Figure 4E; *P* < 0.01). Also, SSc‐MVECs with the overexpression of miR‐126 dramatically enhanced cell migration response to VEGF by 6.52 ± 0.63 folds versus 1.15 ± 0.2 folds in SSc‐MVECs/ctr‐miR/VEGF (Figure 4F; *P* < 0.01).

### 3.6 Reduced expression of miR‐126 in SSc‐MVECs impaired phosphorylation response of ERK and AKT to VEGF

**FIGURE 5:**
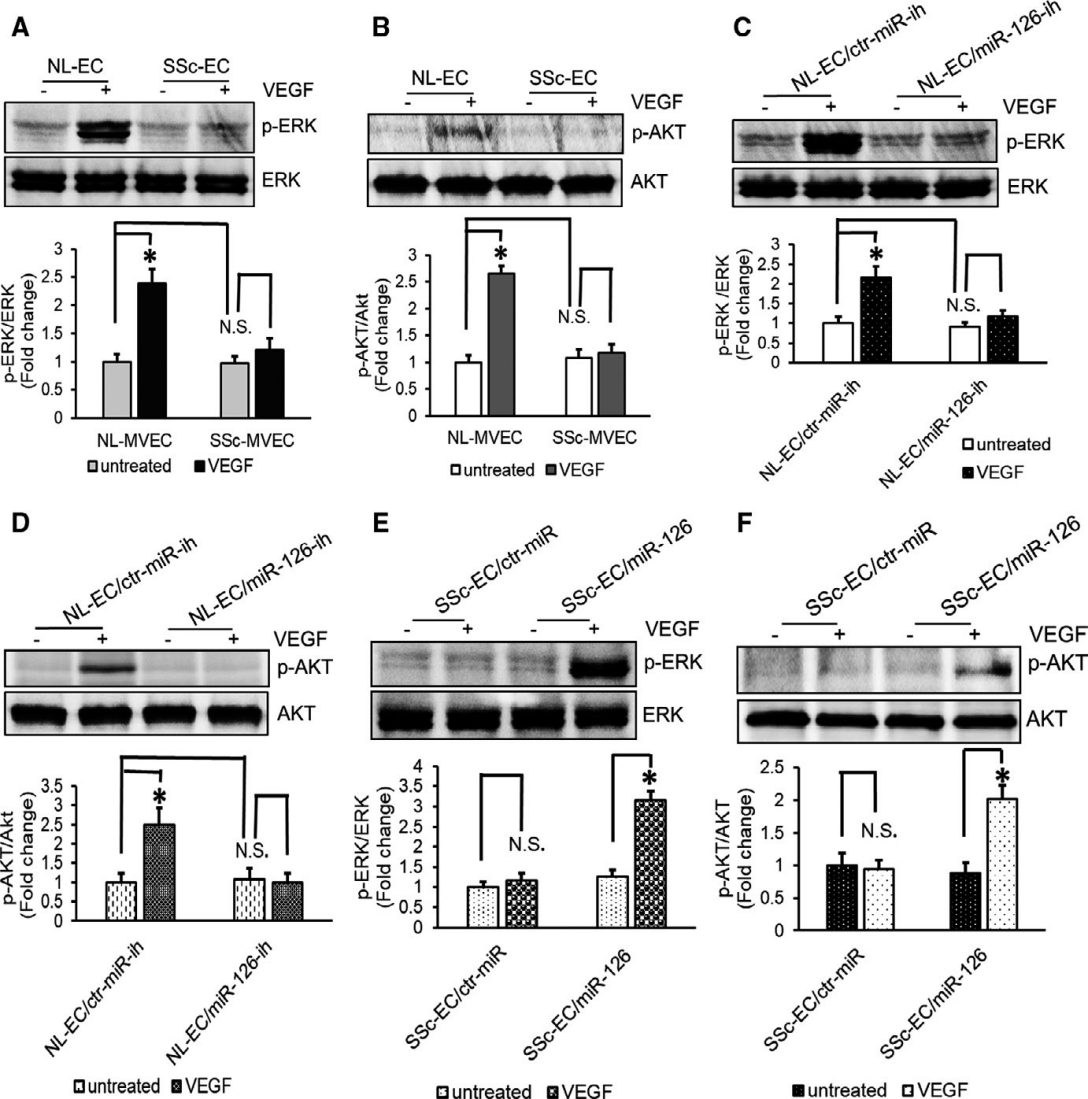
Impaired phosphorylation of ERK and AKT to VEGF in SSc‐MVEC is associated with the reduced expression of miR‐126 The phosphorylated and total ERK1/2 and AKT were assessed by Western blotting. The protein levels were quantitated by NIH ImageJ. The total ERK and AKT were used as the protein loading control for p‐ERK and p‐AKT, respectively. Values are fold change compared to the control without VEGF treatment. ECs were starvation overnight and then were stimulated with and without VEGF 50 ng/ml for 15 minutes. A, B, Diminished activation of ERK and AKT in response to VEGF were seen in SSc‐MVECs, while VEGF significantly increased the phosphorylation of ERK and AKT in NL‐MVECs. C‐D, Knockdown of miR‐126 in NL‐MVECs decreases VEGF‐dependent phosphorylation of ERK and AKT. E‐F, Overexpression of miR‐126 enhances VEGF‐dependent phosphorylation of ERK and phosphorylation of AKT in SSc‐MVECs. *P < 0.01, labelled group versus the other groups. n = 6 different cell lines

To examine the responses of MVECs to VEGF‐induced activation of MAPK and AKT angiogenesis signalling pathways, NL‐MVECs and SSc‐MVECs were subjected to serum and growth factor withdrawal overnight and then subjected to VEGF stimulation at 50ng/ml for 15 minutes. Cell lysates were immunoblotted with the antibodies to determine the level of phosphorylated and total ERK, and phosphorylated and total AKT. Results showed that the VEGF stimulated activation of ERK and AKT in NL‐MVECs, while in SSc‐MVECs minimal activation of ERK and AKT in response to VEGF stimulation was noted (Figure 5A‐B).

To further test if miR‐126 is an essential regulator for phosphorylation of AKT and ERK in response to VEGF, we transfected NL‐MVECs with miR‐126 inhibitor and examined the activation of MAP kinase and PI3 kinase by VEGF stimulation. As shown in Figure 5C‐D, knockdown of miR‐126 expression with miR‐126 inhibitor in NL‐MVECs significantly diminished ERK1/2 phosphorylation and AKT phosphorylation in response to VEGF, compared to microRNA inhibitor control. Conversely, overexpression of miR‐126 by transfection of miR‐126 mimic into SSc‐MVECs dramatically increased ERK1/2 phosphorylation and AKT phosphorylation in response to VEGF by approximately 2.5 folds compared to a microRNA mimic control (Figure 5E‐F).

### 3.7 The reduction of miR‐126 in SSc‐MVECs is linked with the hypermethylation of miR‐126 promoter region

**FIGURE 6:**
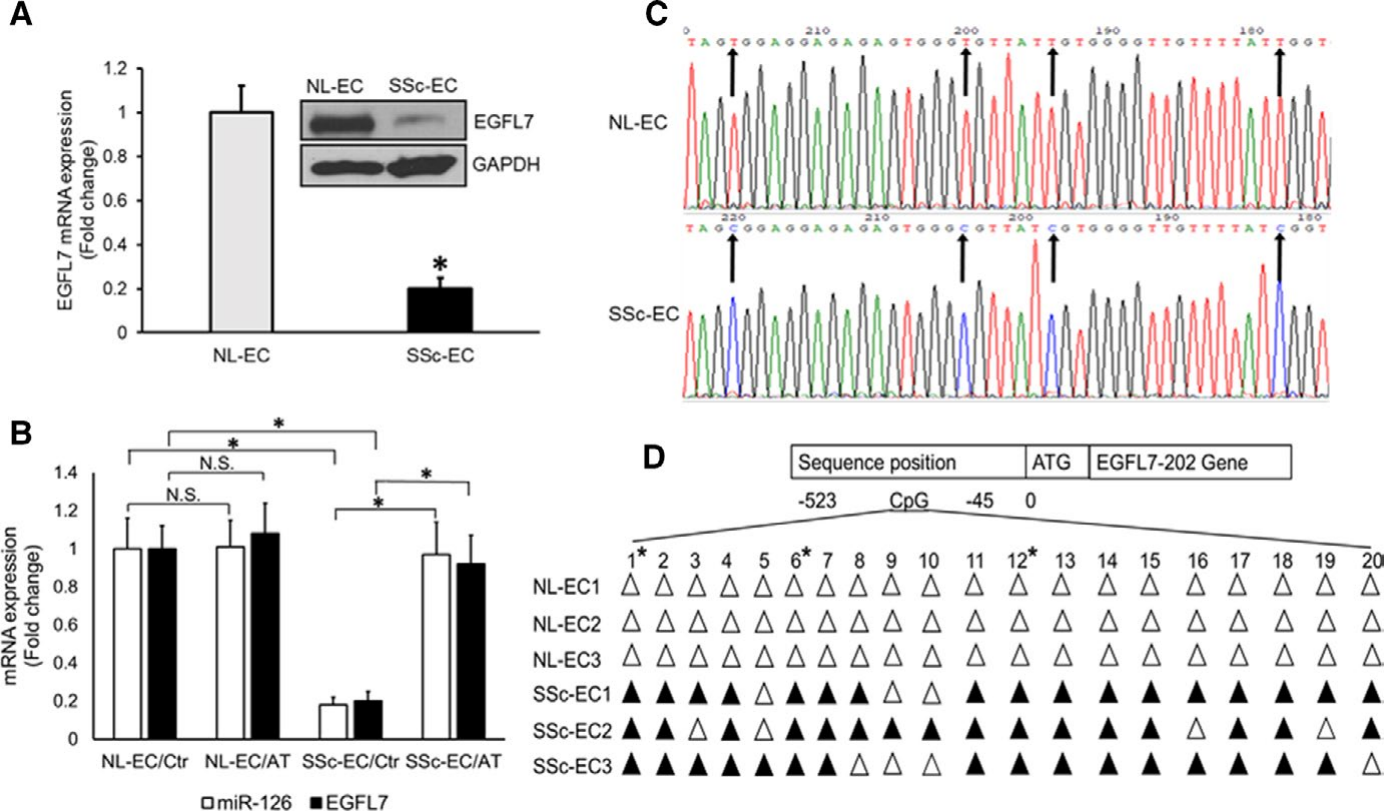
Up‐regulation of miR‐126 expression by epigenetic inhibitor in SSc‐MVECs and hypermethylation of miR‐126 / EGFL7 co‐promoter region in SSc‐MVECs A. Reduced mRNA and protein expression levels of EGFL7 in SSc‐MVECs by qPCR and Western blot. B, Increase mRNA expression of miR‐126 and EGFL7 in SSc‐MVECs using TaqMan real‐time PCR after Aza and TSA treatment. C, Representative sequences of the promoter region of the miR‐126 gene after bisulphite treatment. The arrows indicate CpG sites. After bisulphite treatment, the un‐methylated cytosine (C) is changed to thymine (T), whereas the methylated cytosine is unchanged. D, The methylation status of the CpG sites in the miR‐126 /EGFL7 promoter region (−45 to −523). The cytosine in the CpG sequences −493, −454, −445, −367, −359, −338, −330, −304, −276, −226, −194, −178, −172, −156, −141, −119, −117, −95, −93 and −76 are coded from 1 to 20, respectively. Open triangles indicate no methylation; solid triangles indicate CpG island methylation. Six genomic samples (three from normal endothelial cells and three from SSc endothelial cells) were sequenced. The 1st, 6th and 12th CGs (labelled with *) are within the SP1, Ets1 and EGR transcription binding sites, respectively

To explore the mechanism by which miR‐126 is down‐regulated in SSc‐MVECs, we analysed the expression and the promoter methylation status of EGFL7 gene. MicroRNA‐126 is an intronic microRNA, located within the intron of the EGFL7 locus, and mature miRNA‐126 is produced from the processing of EGFL7 pre–mRNA transcript rather than from its promoter.^25^ Western blot and real‐time PCR analysis revealed that EGFL7 expression was significantly down‐regulated in SSc‐MVECs compared to healthy controls both at mRNA level (*P* < 0.01)) and at protein level (Figure 6A), which were consistent with the down‐regulated miR‐126 expression in SSc‐MVECs (Figure 1C). These results support that miR‐126 and EGFL7 share the same promoter, and their expression levels were controlled by the EGFL7 promoter in MVECs. EGFL7 expression levels may be considered as a biomarker for the miR‐126 expression in tissue.

To investigate whether an epigenetic mechanism mediates underexpression of miR‐126 in SSc‐MVECs, NL‐MVECs and SSc‐MVECs were treated with the DNA methyltransferase inhibitor Aza (5‐Aza‐2′‐deoxycytidine) at 5 µM for 5 days and the histone deacetylase inhibitor TSA (trichostatin A) at 100 nM for the last day. The expression levels of EGFL7 and miR‐126 significantly increased from 0.20 ± 0.05 folds for EGFL7 and 0.18 ± 0.04 for miR‐126 in SSc‐MVEC untreated control to 0.92 ± 0.15 folds for EGFL7 and 0.97 ± 0.16 for miR‐126 in Aza‐ and TSA‐treated SSc‐MVECs (Figure 6B; *P* < 0.01), while no effects on EGFL7 and miR‐126 expression levels were noted in NL‐MVECs after treatment with Aza and TSA (Figure 6B). These data suggest that there is a possibility of epigenetic changes in miR‐126/EGFL7 promoter region in SSc‐MVECs.

To explore the methylation status of the CpG islands within the miR‐126/EGFL7 promoter region, bisulphite genomic sequencing analysis was used in normal and SSc‐MVECs samples. We cloned and sequenced the modified EGFL7 promoter region, −45 to −523 bps upstream of ATG. Dense methylation in the predicted location of CpG islands in the SSc‐MVECs promoter region was noted in DNA derived from three SSc cell lines, whereas no methylation was noted in three matched control cell lines (Figure 6C‐D). These data suggest that the down‐regulation of miR‐126 in SSc‐MVECs is associated with promoter hypermethylation of miR‐126/EGFL7 gene.

To determine if the methylated CpG islands overlap with the transcription factor (TF) binding sites, we used ‘PROMO’, an online software, to examine transcription factor (TF) binding sites in miR‐126/EGFL7 promoter region. There are 20 CpG sites in the sequenced miR‐126/EGFL7 promoter fragment; among them, 8 sites overlap with transcription factors–binding sites. Three of these CpG sites are potentially important in the regulation of EGFL7 transcription. One is a CG‐containing SP1‐binding site, and the other is a CG‐containing Ets1‐binding site. The third one is a CG‐containing EGR‐binding site. All these sites are methylated in all three SSc samples, and they correspond to the 1st, 6th and 12th CGs (Figure 6D). Previous studies reported that transcription factors binding to these sites are essential for the transcriptional regulation of EGFL7,^26^, ^27^, ^28^, ^29^ and therefore, the methylation of these sites of the promoter region can hinder transcriptional factor binding and repress the transcription of EGFL gene and miR‐126 gene in SSc‐MVECs.^30^

## 4 DISCUSSION

The results of this study show that down‐regulation of miR‐126 is associated with impaired SSc endothelial cell responses to VEGF. We observed that the miR‐126 expression levels were decreased in SSc skin and SSc‐MVECs, and when miR‐126 was down‐regulated in NL‐MVECs, the cells lost the ability to mount an angiogenetic response to VEGF, while up‐regulation of miR‐126 restored the VEGF‐induced angiogenic responses in SSc.

Postnatal angiogenesis relies on a proper response of endothelial cells to angiogenic stimuli and experimental evidence points to VEGF signalling as the most powerful angiogenic factor.^6^ Activation of VEGF signalling induces proliferation and migration of endothelial cells in both physiological and pathological angiogenesis. The mechanisms that control VEGF induction, signalling and endothelial cell response remain incompletely understood. Nonetheless, miR‐126 is a crucial post‐transcriptional regulator of MVECs angiogenesis.^15^, ^16^, ^17^, ^31^, ^32^ Targeted deletion of miR‐126 in mice or miR‐126 knockdown in zebrafish resulted in the loss of vascular integrity and defective angiogenesis, while overexpression of miR‐126 regulates angiogenesis in cell‐type and strand‐specific manner.^15^, ^16^, ^31^, ^32^

Computational algorithms predicted that the specific genes, SPRED1 and PIK3R2, are potential targets of miR‐126. It was also reported that miR‐126 regulated and controlled the expressions relevant to other genes including PTPN9, PTEN, SDF‐1, VCAM‐1, HoxA9, v‐Crk and EGFL7. Therefore, miR‐126 plays important role in vascular development, neovascularization, the transition of endothelial progenitor cells to mesenchymal cells, endothelial survival and vascular inflammation.^15^, ^16^, ^31^, ^33^, ^34^, ^35^, ^36^ Further studies demonstrated that miR‐126 can exhibit multiple properties under different conditions. For instance, in foetal development, vessel injury or hypoxia, miR‐126 stimulates angiogenic signalling by targeting VEGF signalling by suppressing SPRED1 and PIK3R2 through activation of the proangiogenic signalling RAF1/Erk1 and PI3K/Akt;^31^, ^37^, ^38^ in addition to targeting SPRED1 and PIK3R2, miRNA‐126 also mediated angiogenesis in the ischaemic mouse brain through direct inhibition of its target, PTPN9 and activation of AKT and ERK signalling pathways;^35^ during burn wound healing, miR‐126 promotes endothelial cell proliferation, migration and angiogenesis and inhibits apoptosis by directly targeting sciellin (SCEL).^39^ Based on these findings, we speculated that miR‐126 potentially regulates the VEGF‐dependent angiogenesis in SSc‐MVECs through targeting SPRED1‐RAF1/Erk1 and PIK3R2‐PI3k/Akt signalling. Consistent with these predictions and studies, our data show the mRNA and protein levels of SPRED1 and PIK3R2 were significantly increased in SSc‐MVECs and after miR‐126 knockdown in NL‐MVECs. Moreover, overexpression of miR‐126 in SSc‐MVECs reduced mRNA and protein expression levels of SPRED1 and PIK3R2.

Phosphorylation of ERK1/2 MAPK, AKT and p38 MAPK by VEGFR2 activation appears to be necessary for stimulation of angiogenesis in endothelial.^7^, ^8^, ^9^ MiR‐126 has been shown to promote MAP kinase and PI3K signalling in response to VEGF and FGF by targeting negative regulators of these signalling pathways, including SPRED1 and PIK3R2.^15^, ^16^ SPRED1 negatively regulates the activation of the MAP kinase pathway by binding and inactivating RAF1, an upstream kinase of the pathway.^40^, ^41^, ^42^ PIK3R2 acts as a suppressor of the PI3K/AKT signalling pathway activation,^33^, ^35^ and knockdown of PIK3R2 rescued the defect in VEGF‐dependent phosphorylation of AKT in miR‐126 knockdown HUVECs.^15^ Consistent with these data, we found that the phosphorylation of ERK1/2 and AKT in response to VEGF is reduced in SSc‐MVECs. Similar responses were also noticed in miR‐126 knockdown in NL‐MVECs, while phosphorylation of ERK1/2 and AKT in response to VEGF was restored to normal levels in SSc‐MVECs after transfection with miR‐126 mimic. These data indicated that down‐regulated miR‐126 in SSc‐MVEC impaired the VEGF‐induced ERK and AKT activation.

MicroRNA‐126 is largely an endothelial‐specific miRNA that is located within intron 7 of EGFL7.^15^, ^16^ MiR‐126 and its host transcript, EGFL7, are highly expressed in endothelial cells. MiR‐126 originates from the EGFL7 pre‐mRNA. Usually, an intronic miRNA tends to be co‐expressed with its host gene.^16^, ^25^, ^43^ A previous study showed that the EGFL7 expression levels were significantly decreased in SSc‐MVECs. EGFL7 transcript knockout mice were reported to display vascular abnormalities remarkably similar to those of miR‐126 null mice,^44^ which suggests that the phenotype of those mutant mice reflects the loss of function of miR‐126. In agreement with previous studies, we observed significantly decreased expression of EGFL7 in SSc‐MVECs compared to the NL‐MVECs. These data suggested that miR‐126 and EGFL7 share the same promoter in MVECs and that underexpression of EGFL7 and miR‐126 in SSc‐MVECs occurs at the mRNA transcription level.

It is known that an epigenetic mechanism is associated with the repression of the gene.^45^, ^46^ Methylation of promoter CpGs is thought to contribute to repression through two mechanisms: (1) direct inhibition of transcription factor binding which is necessary for recruitment of the transcription machinery (represented by RNA polymerase II), and (2) attraction of MeCPs (methyl CpG binding proteins) which associate with co‐repressors such as histone deacetylases.^47^ 5‐aza‐2’‐deoxycytidine, an inhibitor of all DNA methyltransferases that inhibits DNA remethylation after DNA replication.^48^ Gene expression is also regulated by histone acetylation and de‐acetylation. Moreover, our data show some methylated CpG islands overlap with the transcription factor binding sites, including SP1, ERG1 and Ets1 which regulate the expression of EGFL7 and miR‐126 in endothelial cells.^26^, ^27^, ^28^, ^29^ Hypermethylation of miR‐126 promoter in SSc‐MVECs leads to the transcription repression of EGFL7 /miR‐126 gene through obstructing TFs’ binding and the recruitment of MeCP transcription repressing complex.

Some of the limitations of our study include a lack of testing of other angiogenic factors. We also did not evaluate the role of the antiangiogenic VEGF165b splice variant that was reported to be the limiting factor in SSc‐MVECs angiogenic response in SSc.^49^ Also, we did not investigate the relative role of histone alteration in the observed down‐regulation of EGFL7 /miR‐126 gene expression.

In conclusion, our findings show that SSc‐MVECs express reduced levels of miR‐126 and that miR‐126 is required for angiogenesis in SSc‐MVECs. MiR‐126 enhances the VEGF‐induced angiogenesis in SSc‐MVECs by targeting SPRED1 and PIK3R2, which are the negative regulator of Raf‐ERK and PI3K‐AKT signalling separately. Extensive CpG sites’ methylation was found in miR‐126 promoter region in SSc‐MVECs. These results may provide new insights into the pathogenesis of defective angiogenesis and vascular repair in SSc. Administration of proangiogenic miR‐126 or molecular regulation of miRNA‐126 expression might represent potential therapeutic approaches to promote effective angiogenesis and capillary regeneration in SSc.

## CONFLICT OF INTEREST

There are no financial support or other benefits from commercial sources for the work reported on in the manuscript, or any other financial interests that any of the authors may have, which could create a potential conflict of interest or the appearance of a conflict of interest regarding the work.

## AUTHOR CONTRIBUTION

**youngqing Wang:** Conceptualization (equal); Data curation (lead); Formal analysis (lead); Investigation (equal); Methodology (equal); Project administration (supporting); Resources (supporting); Software (supporting); Supervision (equal); Validation (equal); Visualization (equal); Writing‐original draft (equal). **John Sun:** Conceptualization (supporting); Data curation (supporting); Formal analysis (supporting); Investigation (supporting); Methodology (supporting); Software (equal); Validation (equal); Writing‐original draft (equal). **Bashar Kahaleh:** Conceptualization (equal); Data curation (equal); Formal analysis (equal); Project administration (equal); Supervision (equal); Writing‐original draft (equal); Writing‐review & editing (equal).

## Data Availability

The data that support the findings of this study are available from the corresponding author upon reasonable request.
